# Research progress on the role of extracellular vesicles derived from aging cells in osteoporosis

**DOI:** 10.1042/BSR20221775

**Published:** 2023-02-16

**Authors:** Huan Chen, Guowei Huang, Wei Mao, Peiliang He, Guodong Hou, Wencong Zhang, Zhiyi Liu, Aiguo Li, Shengnan Qin

**Affiliations:** 1 Department of Orthopedics, Guangzhou Institute of Traumatic Surgery, Guangzhou Red Cross Hospital, Medical College, Jinan University, Guangzhou, China; 2 School of Biomedical Science, The University of Western Australia, Perth, Australia

**Keywords:** Aging, Extracellular vesicles, Osteoporosis, Senescence-associated secrete phenotype

## Abstract

The occurrence and development of many diseases are highly associated with the aging of the body. Among them, osteoporosis (OP) is a common age-related disease that tends to occur in the elderly population and is highly related to the aging factors in the body. In the process of aging transmission, the senescence-related secretory phenotype (SASP) can convey the information about aging through the paracrine pathway and endocrine mechanism through the extracellular vesicles (EVs) connected to SASP. EVs can be used as a way of conduction to join the connection between micro-environmental aging and age-related illnesses. EVs are double-layer membranous vesicles separated or secreted from the cell membrane, which mainly include microvesicles (MVs) and exosomes. Vesicular bodies secreted by this exocrine form carry a variety of cell-derived related substances (including a variety of proteins, lipids, DNA, mRNA, miRNAs, etc). These substances are mainly concentrated in human body fluids, especially can be transported to all parts of the body with the blood circulation system, and participate in the interactions between cells. Osteoporosis is closely associated with aging and aging cells, suggesting EVs were active in this pathological process. In this article, the basic mechanisms of aging cells in the occurrence and progression of osteoporosis through EVs will be discussed, to explore the connection between aging and osteoporosis, thereby providing a new perspective on the occurrence and development as well as prevention and treatment of osteoporosis.

## Introduction

The loss of balance in bone remodeling is what defines osteoporosis, which is known as the process of skeletal change and entails the resorption of old and damaged bone mediated by osteoclasts, followed by the deposition of new bone regulated by osteoblast lineage cells. Osteoporosis occurs when bone resorption outweighs bone formation, resulting in the decreased bone mass, destruction of bone microstructure, and increased bone fragility. Fractures occur easily by very minimal trauma, like cough, in patients who are suffering from osteoporosis. It is expected that there will be one osteoporotic fracture in every two fractures in the 2050s [1].

The intercellular communication between but not limit to osteoblasts and osteoclasts plays an essential role in osteoporosis as osteoblasts and osteoclasts primary occupy discrete territories without physical interaction in the steady-state in bone remodeling [2], and there is a time delay of several weeks between the completion of bone resorption and the commencement of bone formation [3]. In this process, any change of the intercellular communication might induce the imbalance of bone homeostasis. Mutual communication between osteoblasts and osteoclasts without contact can be achieved in several ways, including secreted growth factors and extracellular vesicles (EVs). Among this, osteoclast-derived EVs have been proven the regulatory factor of osteoclastogenesis and osteoblast–osteoclast communication in skeleton diseases [4].

EVs, a kind of small membrane vesicles 30–100 nm in diameter, are secreted by cells and released into extracellular spaces. EVs are one of the most crucial intercellular communication mediators because they can transmit their cargo, which includes lipids, nucleic acids, and proteins, to nearby or distant cells, changing the physiological activity of recipient cells. EVs are common in serum and other bodily fluids [5,6].

Osteoporosis is a senile disease highly associated with hyperactive senescent cells. Among the hallmarks of senescence, the senescence-associated secretory phenotype (SASP), in particular, SASP-related EV signal transduction is mainly carried out through paracrine and endocrine ways [7].

In this review, we detail the production of EVs and their role in information transmission from aging cells in the osteoporosis and hope to provide new ways for anti-osteoporosis treatment with aging-related EVs.

## Osteoporosis and factors affecting bone remodeling

Osteoporosis, as the most common bone-related diseases in clinic, is demonstrated by the lower bone density, porous bone microstructure, and thinner bone trabeculae than normal ones. Osteoporosis mainly includes primary osteoporosis and secondary osteoporosis. Primary osteoporosis includes postmenopausal osteoporosis, senile osteoporosis, idiopathic osteoporosis, and so on, while the second one refers to the disease of bone loss resulting from the external use of drugs and related diseases.

Senile osteoporosis is bone loss that results from aging [8]. It may cause no symptoms at first, and most of cases are found because of the occurrence of fractures. With the rapid increase of the aging population, the prevalence of senile osteoporosis is increasing year by year [9]. It is reported that the morbidity of osteoporosis in the elderly (≥50 years old) in China would increase from 15.77% (95% CI: 8.91–25.98%) in 2020 to 23.43% (95% CI: 14.51–35.26%) in 2050 [10]. Given the high correlation between senile osteoporosis and aging, this article emphasizes on senile osteoporosis.

Bone tissues serve as a repository for vital minerals, including calcium phosphate and various biologically active molecules, such as growth factors. Osteoclasts are mobile on the bone surface and responsible for liberating minerals and other molecules stored within the bone matrix, while osteoblasts/osteocytes are responsible for forming new bone matrix to maintain the bone mass. In the adult skeleton, osteoblasts/osteocytes, which control bone creation, maintain a delicate balance with osteoclasts, which control bone resorption. Osteoporosis occurs because of the long-standing imbalance between bone formation and bone resorption.

**Figure 1:**
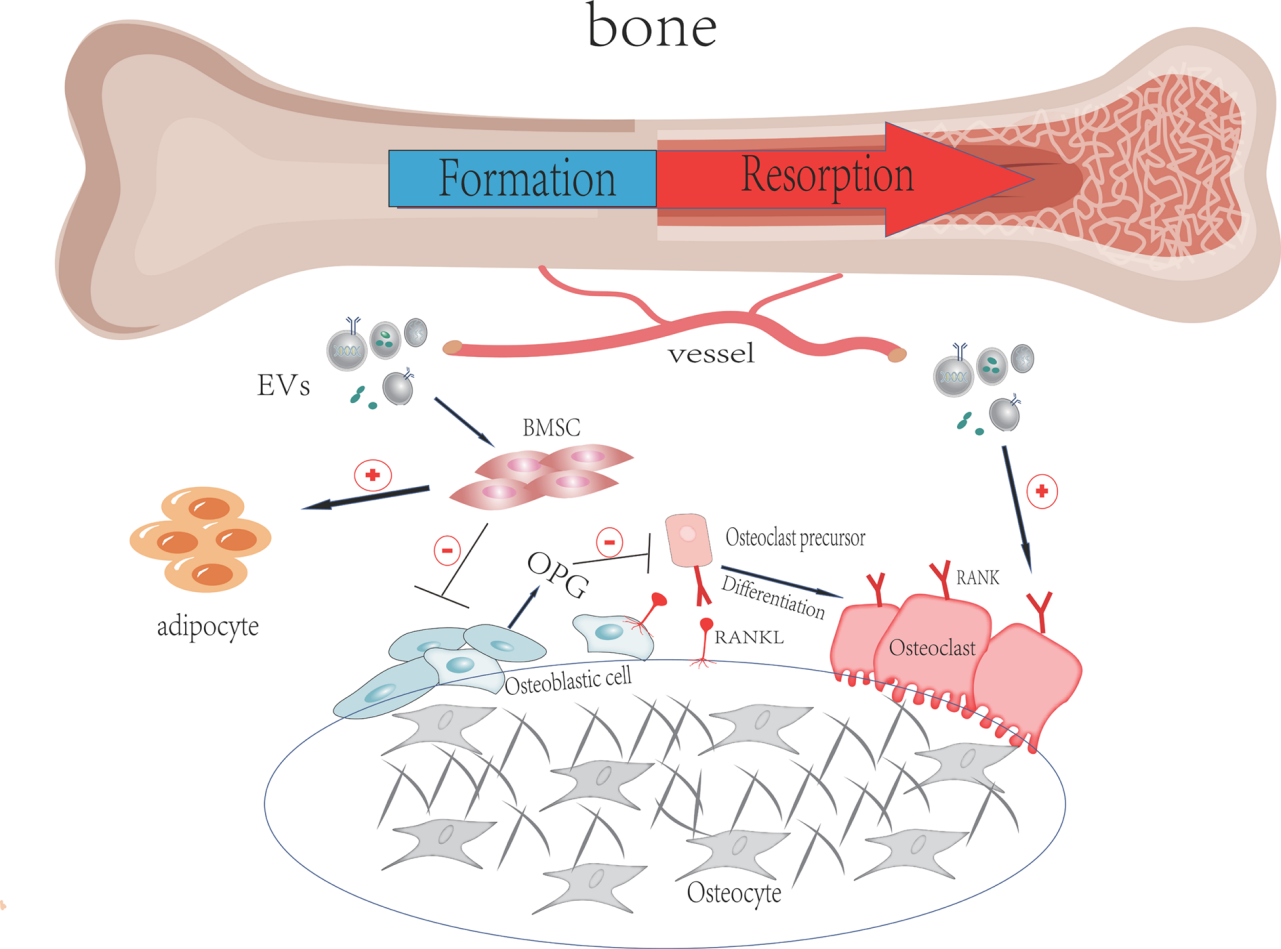
The figure shows the balance of the bone microenvironment, mainly the balance between bone absorption and bone reconstruction The expression of RANK on osteoclast precursor cells further differentiated into osteoclasts under the combination with RANKL. As the main source of OPG in bone regulation, osteoblasts can produce RANKL and OPG to control the generation of osteoclasts. When the EVs carrying ‘carriers’ reach the bone microenvironment, the balance between osteogenesis and osteoclast may be destroyed.

By now, there is no clear pathogenesis for the occurrence and development of senile osteoporosis. Researchers mainly focus on the functional disorder of osteoblasts and osteoclasts and the intercommunication between different types of cells [11,12]. Among it, the intercellular communication between osteoblasts and osteoclasts plays a vital role in maintaining bone homeostasis [13]. Osteoclasts (OCs) can deliver many signals to direct and coordinate the behavior of osteoblasts (OBs), and vice versa. Mutual communication between OBs and OCs without contact can be achieved in several ways, including secreted growth factors [3] and EVs [4]. For example, M-CSF and RANKL are essential for osteoclastogenesis. M-CSF binds to its specific receptor C-FMS on the surface of osteoclasts and monocytes/macrophages after being released by osteoblasts and bone marrow stromal cells. The expression of RANKL has a high level in osteoblasts and osteocytes [14]. RANKL and NF-κB (RANK) receptor activator leads to the production and differentiation of osteoclasts in which RANK is mainly expressed on the surface of osteoclasts and its precursors [15]. Osteoprotegerin (OPG) is also secreted from osteoblasts negatively regulating osteoclast formation by destruction the RANKL–RANK interaction as a decoy receptor of RANKL [16] (Figure 1).

In the generation and development of senile osteoporosis, SASP-related EV signals play a major role in spreading aging through paracrine and endocrine mechanisms [7]. Successful communication between the EVs and the aging milieu and age-related disorders causes negative effects on immunization, inflammation, genetic expression, and metabolism at the level of cell information transmission, as well as limiting the downstream signal pathway.

## Specificities of secretion and uptake of EVs for cell-to-cell communication

### Source and definition of EVs

**Figure 2:**
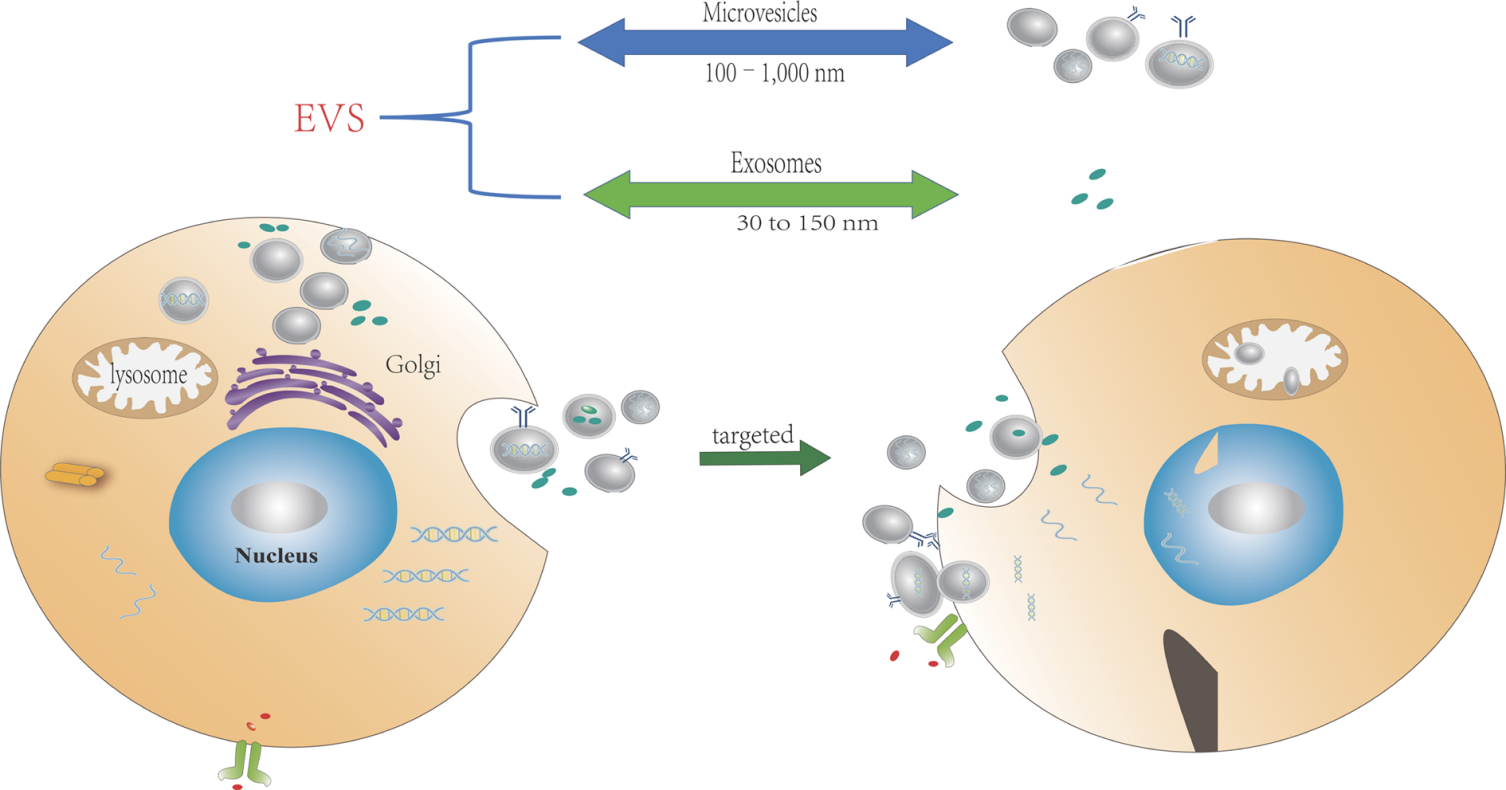
This is a figure of cells secreting EVs with double membrane structures By targeting the receptor cells or through nonspecific macropinocytosis or micropinocytosis, these vesicles, used as ‘carriers’ for communication between cells, can carry proteins, lipids, nucleic acids, and other bioactive molecules to transmit signals between cells. Once the EVs enter the target cells, they can directly release the bioactive molecular goods carried into the cytoplasm, or they can be recovered and re-secreted or degraded by lysosomes.

The International Society of Extracellular Vesicles (ISEV) believes that two distinct isoforms of EV have been widely confirmed based on the mechanism of EV biological origin: one is from the protoplasm membrane (microvesicles, ectosomes, and microparticles), and the other is from the inner system/polyvesicles (MVB) (exosomes) [17]. Microvesicles (MVs), exosomes, and apoptotic bodies are the three primary hypotypes of EVs, and they are categorized according to their release mechanisms, size, composition, and function [18]. So far, no specific protein marker has been determined to distinguish different types of EVs because they are formed in different ways and have different protein mass spectra [19]. The size of vesicles is usually used for characterization. The size of MV ranges from 50 to 10,000 nm, and the exocrine body is small, with a diameter of 30–150 nm. In general, electric vehicles include a variety of vesicles ranging in size from 30 to 1000 nm, with various types of bioactive substances, and different types of vesicles have overlap in their size distribution (Figure 2).

### The release and uptake of extracellular vesicles

The generation and release of different types of EVs are also different due to the different intracellular events and this process has been well reviewed [20]. In short, once the MV is formed, it will be separated from the plasma membrane, and the exocrine body needs multivesicular endosomes (MVEs) to transport and attach to the plasmalemma, so as to fuse with the exocrine body and unleashed on the extracellular space. Owing to the difference in secretion, microvesicles would be released faster than exosomes, since the targeting of cargoes to microvesicles merely requires that they remain at the plasmalemma, and the release of those cargoes would occur immediately after the microvesicles’ formation and fission. However, releasing exosomes require more procedures, because the goods are classified as MVE and then classified into exosomes, as well as the additional steps of targeting MVE to the plasma membrane and initiating their release.

Although the content and volume of EVs are different, the uptake of different subsets of EVs and the general principle of intercellular transport may be the same [21]. Once released into the extracellular space, EVs are uptook by recipient cells and undergo different fates. The cargoes of EVs are delivered to the recipient cells whose phenotypes changed, and subsequently their physiological or pathological status were affected. There are three different EV-mediated intracellular communication: docking at the plasmalemma, activated surface receptors and signaling, liposome internalization or synthesis with target cells. For docking, EVs can only dock on the plasmalemma or limiting membrane of MVE, and then release its goods (such as microRNA) into the cytoplasm of recipient cells through fusion. For the activation of receptors, EV can bind to the surface like integrin, thus initiating intracellular signal pathways, such as antigen presentation [22]. For internalization, EVs may be internalized by multiple pathways but the mechanisms underlying it are not fully unraveled [21]. Many different processes, such as clathrin-mediated endocytosis (CME), phagocytosis, macropinocytosis, and plasma or endosomal membrane fusion, have been postulated for EV absorption [23]. Under the influence of special factors, such as ‘pressure stimulation’, cells can specifically design some EVs related to them and carry active substances (ti-RNA or active protein). These EVs can be enhanced by specific cells to absorb, release the active substances in the EVs, and complete a unique niche regulation mode. Therefore, the transfer of active substances in EV cells provides a stress modulation signal axis different from the traditional cytokine driven stress response [24].

## Aging, cell senescence, and extracellular vesicles

Aging is a nonreversible sophisticated physiological process and exists throughout the whole life. Age-related disorders and cell senescence, a state of sustained hyperactivity in an aging environment, are strongly related [7]. Evidences also showed that senescent cells were involved in the development of osteoporosis [25–27].

Cellular senescence is a stage between life and death that tightly controls cellular survival and demise. Senescence cell elimination is crucial for encouraging tissue healing, controlling organ development, and maintaining body homeostasis [28,29]. In diseases related to aging, the excessive accumulation of aging cells in the process of aging destroys the track of programmed cell death and leads to the regrowth declined [30]. Senescent cells have three main characteristics such as cell proliferation arrest, apoptosis resistance, and complex senescence-associated secretory phenotypes (SASP) [31]. A lot of senescent cells can be found in senescent and diseased tissues of many species [32], and can promote a variety of aging phenotypes and serve as markers of disease, mainly through the nonautonomous effect of aging-related secretory phenotypes, *in vitro* [33]. Transgenic mouse models that selectively scavenge senescent cells and their secreted SASP have also confirmed that aging cells had a causal connection with a lot of age-related diseases *in vivo* [34,35].

SASP as the sign of senescence, SASP-related EV signal transduction plays a significant part in the transmission of aging[7]. As a successful way of information exchange, EVs exist in the micro-environmental aging and age-related illnesses [7].

The senescence-associated secretory phenotypes are formed after cell aging, which can affect cell function through the release and *in vivo* circulation of EVs [36]. After EVs secreted by aging cells enter the cells, the SASP-like bioactive substances in them come into effect [37]. These SASP-like bioactive substances involve a series of functions, including intercellular adhesion, cell signal transduction, leukocyte cross-endothelial migration, and antigen presentation [38]. Once this process occurs, it will cause a series of changes in normal biological processes, and lead to the disorder of the cell microenvironment, eventually resulting in the occurrence of aging-related diseases.

## Relation between aging, extracellular vesicles, and bone metabolic balance

Cell senescence plays a key role in the pathogenesis of many age-related illness, including OP, Type 2 diabetes mellitus (T2DM), metabolic syndrome, and possibly other endocrine-related diseases [39]. In osteoporosis-related studies, it has been reported that the elimination of aging cells in mice could reduce bone resorption and increase bone formation and maintenance [40,41].

### Extracellular vehicles showed the possibility to regulate bone homeostasis

The dynamic balance between osteoblasts and osteoclasts is the basis for maintaining the homeostasis of bone homogeneity, while EVs are vesicles of endocytosis origin between cells, which can mediate the process of intercellular communication and participate in the balance of osteoblasts and osteoclasts. Exosomes secreted by MSCs from the neonatal umbilical cord (UC) could enhance the regenerative capacity of aging adult bone marrow-derived MSCs in bone formation, wound healing, and angiogenesis [42]. Pepe et al. [43] demonstrated much more EV and RANKL^+^EVs in osteoporosis patients than osteopenia patients and healthy individuals, and osteoporosis EVs promoted osteoclastogenesis and inhibited osteogenesis *in vitro*, suggesting EVs from osteoporosis could regulate bone homeostasis.

Moreover, micro-RNAs could shuttle between cells and tissues through EVs, enabling them to reach distant cells and play a systemic role. EVs with miR-20a incorporated could directly regulate the osteogenic differentiation of bone mesenchymal stem cells (MSCs) [44], and miR-20a was down-regulated in elderly individuals [45], indicating that EVs rich in miR-20a might be highly correlated with aging process. miR-152-3p, rich in small tumor-derived EVs (SEV), is a potential osteolytic active substance, and targets osteoclasts and cause osteolytic changes [46]. Davis et al. [47] found that EVs from either young or old mice were rich in miRNAs, but the miRNA spectrum of EVs derived from the bone marrow was different at different ages, and the miR-183 cluster (miR-96/-182/-183) was expressed strongly in old EVs. In the meantime, the osteogenic development of BMSCs from young mice may be inhibited by aged EVs. MiR-183-5p mimic transfection of BMSCs may inhibit osteogenic differentiation and cell multiplication, which may hasten aging. In addition, aged muscle-derived EVs also had effects on bone homeostasis via their microRNA cargo. Fulzele et al. [48] found that aged muscle-derived EVs had elevated levels of miR-34a, and decreased viability of BMSCs and increased BMSC senescence.

### Extracellular vehicles are involved in bone homeostasis in aging-transmission

Senescent cells use SASP, which involves EVs, to transmit senescence-related active substances, thus promoting the aging process of cells and organs [49]. In the aging environment, as a component of SASP, EVs have undergone significant changes in aging cells [7]. These EVs promote the delivery of aging by carrying destructive signals in senescent cells and being secreted by senescent cells, and then contacting with target cells, leading to aging of target cells. Briefly, senescence-related EVs secreted from the protoplasm membrane and inner body system can increase DNA and mitochondrial damage with the growth of age, and transmit aging signals to amplify the aging signals between cells and organs. Senescence-associated EVs can induce the aging of target cells through various biological process changes closely related to aging, including immune and inflammatory activation, epigenetic changes, mitochondrial damage, lysosomal system dysfunction, ROS amassed, and stem cell collapse [7]. For instance, in the DNA damage-induced aging, p53 and p65 have the functional role in the production of EVs. The levels of CD63+EVs can be decreased by inhibiting p53, thereby affecting the senescence and apoptosis of adjacent cells [50]. Age-induced inflammatory information can be transmitted in mesenchymal stem cells through EVs, and the transmission of this information can be reduced by inhibiting p65 signal pathway, which shows the criticality of NF-κB in the SASP [51].

### Extracellular vehicles from senescent cells involved bone homeostasis

BMSCs could differentiate into osteoblasts and promote osteogenesis, playing an important role in maintaining bone mass [52]. However, with the aggravation of the aging process, SASP-related active factors secreted by aging cells accelerated the aging process of BMSCs, which lead to the decline of osteogenic ability and the imbalance of bone metabolism, and ultimately osteoporosis occurred [53].

According to numerous reports, aging-related disorders are intimately related to the senescence of BMSCs, such as osteoporosis. Liu et al. [54] found that leucine-rich repeat-containing 17 (LRRc17) expression in BMSCs showed a strong positive correlation with aging, and LRRc17 knockdown regenerated senescent BMSCs and improved their therapeutic efficacy in osteoporosis via activating autophagy. In addition, as observed in age-related osteopenia, the fat in bone marrow space gradually accumulated with age, and adipogenesis was considered to be an important indicator of bone tissue aging. Both adipocytes and osteoblasts in the bone marrow were derived from BMSCs, so the reduction of osteoblast to osteocyte transformation may be caused by excessive differentiation into adipocytes during the aging process of BMSCs [55]. During the process of aging, the bone loss could be caused not only by the enhancement of bone resorption activity but also by the dysfunction of MSCs, which would lead to the excessive differentiation of BMSCs into adipocytes [56]. Aged bone matrix-derived EVs (AB-EVs) during bone resorption boost BMSC differentiate toward adipogenesis rather than osteogenic differentiation and increase vascular smooth muscle cell calcification [57]. Alendronate (ALE), a bone resorption inhibitor, also inhibits the release of AB-EVs and lessens the bone-fat imbalance brought on by aging and ovariectomy. It has been discovered that miR-483-5p and miR-2861 are profuse in AB-EVs and are crucial for the AB-EVs-induced imbalance of osteogenesis and adipogenesis, also worsening of blood-vessel calcification.

## Summary and outlook

**Figure 3:**
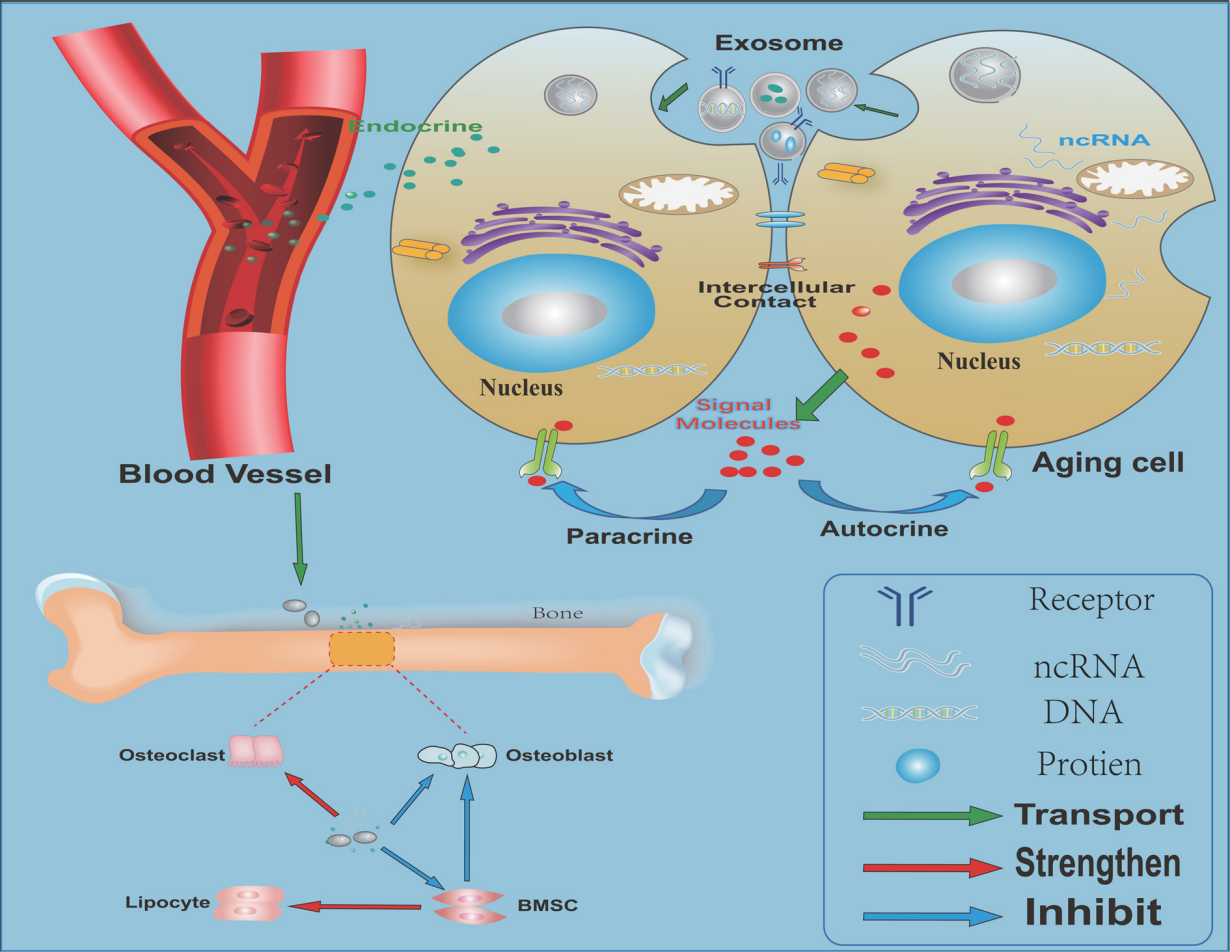
Relation between senescent cells and their secretory extracellular vesicles in bone remodeling EVs are mainly composed of bioactive substances such as MVs, exosomes, proteins, lipids, and noncoding RNAs (ncRNAs). The EVs secreted by senescent cells act as SASP, which can induce senescence of surrounding cells through direct contact between cells and can also reach all parts of the body through blood circulation. The blood supply in the bone is abundant. This kind of SASP-like EVs will damage the balance of the microenvironment in the bone. The aging of BMSCs in the bone will reduce their osteogenic differentiation ability, enhance the differentiation of adipogenic cells, promote the differentiation of osteoclasts, and cause bone loss.

Based on the facts mentioned above, we come to some conclusions about the connection between senescent cells and their secretory EVs in bone remodeling (Figure 3). First of all, aging is an irreversible biological behavior that occurs in various tissues of the body. SASP-related active factors secreted by senescent cells change the microenvironment in bone, affect the activity of BMSCs, reduce their function and differentiation, and enhance adipogenic differentiation. The EVs secreted by aging cells increases and rich in SASP factors, can enter the bone marrow microenvironment with blood flow, and have an impact on the bone marrow microenvironment, and hence affect the behaviors of osteoblastic and osteoclastic lineage cells.

Secondly, the aging signals could be transmitted by SASP involving EVs. The occurrence of aging can promote the secretion of EVs, transfer the aging active factors in the vesicles to the recipient cells, accelerate the aging process, and affect cell function. The exosomes and the ‘active substances’ carried by them can work in the ‘aging-related secretory phenotype’ of aging cells and provide a carrier and way for signal transmission between cells. In addition, the ‘letters’ carried by EVs secreted by various types of cells are different, suggesting EVs from different aging cells had different roles in bone homeostasis.

At last, aging is an important factor in vascular endothelial dysfunction. In the intravascular environment, aging may lead to the reduction of intravascular neovascularization, the slowing down of repairing bone damage, and the serious bone loss, and finally osteoporosis occurs.

Osteoporosis is characterized by bone mass loss, but the key affecting bone mass remains poorly understood but highly associated with aging. The EVs secreted by senescent cells provide a new idea for the study of the functional changes of BMSCs, osteoblasts, and osteoclasts, and also put forward the information exchange between cells, that is, through cell vesicle transport, which combines the cells of the whole body. The EVs of senescent cells also act as SASP-related active factors and play a key role in aging-related diseases represented by osteoporosis. Therefore, clarifying the relation between senescent cells and their EVs and the way they affect cell function may provide a new treatment strategy for aging diseases such as osteoporosis.

## Abbreviations

AB-EV: aged bone matrix-derived EV
ALE: alendronate
BMSC: bone marrow stromal cell
CME: clathrin-mediated endocytosis
EV: extracellular vesicle
HMOX1: heme oxygenase 1
lncRNA: long noncoding RNA
LRRc17: leucine-rich repeat-containing 17
M-CSF: macrophage colony-stimulating factor
MSC: mesenchymal stem cell
MV: microvesicle
MVE: multivesicular endosome
ncRNA: noncoding RNA
OB: osteoblast
OC: osteoclast
OP: osteoporosis
OPG: osteoprotegerin
OVX: ovariectomy
SASP: senescence-associated secretory phenotype
SEV: small tumor-derived EVs
T2DM: Type 2 diabetes mellitus
UC: umbilical cord
